# The efficiency of different types of extenders for semen cooling in stallions

**DOI:** 10.5713/ab.21.0300

**Published:** 2022-01-05

**Authors:** Zuzana Rečková, Radek Filipčík, Katarína Soušková, Tomáš Kopec, Martin Hošek, Vojtěch Pešan

**Affiliations:** 1 Department of Animal Breeding, Mendel University in Brno (FA). Zemědělská 1, 613 00 Brno, Czech Republic

**Keywords:** Fertilizing Capacity, Semen Cooling, Semen Extender, Stallion

## Abstract

**Objective:**

This study was conducted to examine influence of skimmed milk-based extender (SM), INRA 96 extender and BotuSemen Gold extender on parameters of stallions’ ejaculate during storage.

**Methods:**

In this study, 14 stallions between 4 and 20 years of age were monitored. Total and progressive motility, viability and morphology of sperm were evaluated at time intervals of 24, 48, and 72 hours after collection.

**Results:**

The total motility, progressive motility, and values of sperm with normal morphology were significantly higher in the INRA 96 and BotuSemen Gold extenders than in the SM (p<0.01). The sperm viability differed significantly in all extenders (p<0.01). The highest value of sperm viability was in INRA 96 (64.69%±0.67%) and lowest in SM (59.70%±0.81%). The highest differences occurred at 72 hours of storage. Values of total motility, progressive motility and sperm viability decreased over time (p<0.01). In case of sperm morphology there was no statistically significant decrease between 48- and 72-hour time intervals.

**Conclusion:**

It can be concluded that the extenders with a chemically defined composition have shown better indicators of insemination capabilities in ejaculates than the SM. The BotuSemen Gold extender is a suitable alternative to the INRA 96, when used within 48 hours; after 72 hours of storage, however, the INRA 96 showed a higher share of viable spermatozoa.

## INTRODUCTION

Artificial insemination is currently a widespread biotechnological method that significantly influences genetic progress in equine industry due to the subsequent increased availability of the best stallions [1]. Cooled insemination doses are used more often than frozen ones and show better indicators of insemination capability of the semen used [2]. However, its disadvantage lies in the limited available storage period. Most extenders guarantee quality of an ejaculate for 24 hours. After this period, the liveability of the sperm decreases [3]. The sperm liveability is mainly influenced by the composition of the extender [4].

Over time, a variety of extenders for equine semen cooling have been developed [5]. Usually, diluents are comprised of components such as sugars, electrolytes, buffers, egg yolk, milk, and milk products [6]. Sugars such as glucose enhance sperm motility during semen storage [7]. A proper ratio of electrolytes and nonelectrolytes in the extender is important for maintaining the optimal osmotic pressure and the buffering agents in extenders play a key role in prolonging the sperm motility. Egg yolk, milk and milk products protect spermatozoa against temperature changes, especially cold shock [8].

One of the most used diluents in cooled semen technology is skimmed milk-based extender (SM) [6] which represents an effective and practical way of short-term preservation of stallion semen [9]. Milk, being a biological product with a complex composition, is comprised of substances beneficial to spermatozoa, as well as components that can harm their longevity. Milk fractions like α-lactoglobulin negatively affect sperm motility; on the contrary, β-lactoglobulin and native phosphocaseinate are beneficial to spermatozoa [10]. For this reason, extenders with chemically defined compositions have been created. These extenders consist of substances demonstrably beneficial to sperm and reduce potential inconsistency of separate batches compared to the use of skim milk [5,9]. One of these extenders is INRA 96 which is based on modified Hanks’ salts, supplemented with native phosphocaseinate [6]. Native phosphocaseinate is a milk component which consist of α, β, and κ caseins [10] and displays a direct protective effect on stallion spermatozoa [11]. INRA 96 has been the superior and the most often used extender for a long time [3,12]. Lately, a new extender has been introduced to the market, BotuSemen Gold. This extender contains sodium caseinate combined with cholesterol-loaded cyclodextrin [12]. Casein micelles are active components acting as semen protectors [13]. The semen protection mechanism is based on binding to seminal plasma proteins and reducing the lipid loss from the sperm membrane during semen storage [14]. The cholesterol in the BotuSemen Gold extender intervenes using the cyclodextrin in the plasmatic membrane of semen, which stabilises it and thus influences the insemination capability of the dose [15].

In the preliminary study, we tested the semen quality after using SM and commercially available extenders with chemically defined composition Equi Pro, Equi Plus, INRA 96, and BotuSemen Gold. Skimmed milk-based extender, INRA 96 and BotuSemen Gold achieved best results, where the skimmed milk extender performed better than some of the novel diluents [16].

Studies comparing INRA 96 with other chemically defined and milk-based extenders up to 72 hours and INRA 96 with novel extender BotuSemen Gold when stored up to 48 hours have been published [3,12]. The aim of our study was to compare the effectiveness of a SM which is practical and represents an opportunity to reduce costs associated with the production of insemination doses [17] and some of the best commercially available extenders with chemically defined composition, INRA 96 and BotuSemen Gold [12] storing up to 72 hours.

## MATERIALS AND METHODS

### Animals and semen collection

Within the study, 14 stallions of various breeds commercially used for artificial insemination ranging from the age of 4 to 20 years old were monitored. The stallions were stabled in the Tlumačov Regional Stud Farm (Zlín Region, Czech Republic). The semen was collected regularly, once a week, during the breeding season (July to August 2020). The semen collection was executed in the Reproduction Centre of Tlumačov Regional Stud Farm (Czech Republic) using an artificial Missouri vagina (Minitübe GmbH, Tiefenbach, Germany). The study was conducted according to the guidelines of the Declaration of Helsinki and approved by The Ethics Committee of Expert Commission for Ensuring the Welfare of Experimental Animals of Mendel University in Brno (protocol code 16OZ27083/2014-17214 and date of approval 20 May 2019).

The total number of ejaculate samples was 57. Number of collections from each stallion was 3 to 5. A minimal gel-free volume was 15 mL, minimal concentration was 90×10^6^ spermatozoa/mL, and minimal total motility was 60%. All collected ejaculates were used within the study.

### Chemicals and extenders preparation

The SM was prepared based on the recipe of the Tlumačov Regional Stud Farm. The SM extender contains skimmed milk powder, glucose, distilled water, and gentamycin. The BotuSemen Gold extender was supplied by the Nidacon International AB company (Nidacon, Mölndal, Sweden). BotuSemen Gold contains caseins, sugars, amino acids, antioxidants, cholesterol, and supplementary substances. Before the collection itself, the BotuSemen Gold extender was diluted with sterilised distilled water. The INRA 96 extender was supplied by the IMV Technologies company (IMV, L’Aigle, France). INRA 96 contains caseins, puffers, salts, sugars, ultra-pure water, and antibiotics (penicillin sodium, gentamycin sulphate, and amphotericin B). The exact chemical composition of extenders is a trade secret of the producers.

A 25% solution of glutaraldehyde (Sigma, Saint Louis, MO, USA) was used for fixation of the samples for evaluating sperm viability. A Hoechst 33258 (Sigma, USA) was used for dyeing of the samples. Before use, the 25% solution of glutaraldehyde was diluted with sterilised water to a 2.5% solution of glutaraldehyde. The Hoechst 33258 dye was diluted with sterilised water to a concentration of 10 mg/mL.

### Semen samples

Immediately after collection, the gel fraction was removed from the ejaculate by filtering it through sterile gauze. Then, the gel-free semen volume was determined, as well as the concentration, and total and progressive motility of sperm. Smears for evaluation of sperm morphology were made from native ejaculates and samples to determine the sperm viability; these were taken and conserved with a 2.5% solution of glutaraldehyde in the ratio of 1:1 [18].

Three samples were made from each ejaculate. Each sample of the volume of 2 mL was mixed with 4 mL of an extender. The ratio of dilution was 1:2 and total volume of samples was thus 6 mL. One sample (S1) was diluted with the SM, the second one (S2) was diluted with the INRA 96 extender and the third sample (S3) was diluted with the BotuSemen Gold extender.

After 24, 48, and 72 hours after the collection, the total and progressive sperm motility of ejaculates diluted with the SM, the INRA 96 extender and the BotuSemen Gold extender was determined. Samples for determination of the spermatozoa viability were taken from the diluted ejaculates and were mixed with the 2.5% glutaraldehyde solution in a 1:1 ratio [18]. Additional samples were taken from diluted ejaculates and were used for preparation of smears for sperm morphology evaluation. The samples were stored at 4°C.

### Semen samples evaluation

The concentration, total and progressive motility of sperm was determined with the use of the Sperm Class Analyzer CASA system (Microptic SL, Barcelona, Spain) and the Nikon Eclipse E200 microscope (Nikon Instruments Inc., Melville, NY, USA) at 37°C [19]. At least 500 spermatozoa were evaluated in a minimum of five fields of view.

The sperm viability was evaluated using fluorescence microscopy. The ejaculate sample was mixed with the Hoechst 33258 dye in a1:1 ratio and then evaluated using the Olympus BX51 fluorescence microscope (Olympus Corporation, Tokyo, Japan) at 400× magnification [18]. The minimum number of evaluated spermatozoa was 200.

Smears for the morphological evaluation of the sperm were dyed using the Farelly method and evaluated through the Euromex BioBlue microscope (Euromex Microscopen bv, Arnhem, Netherlands) at 1,000× magnification using immersion oil. The minimum number of evaluated spermatozoa was 200. The abnormalities evaluated at this step were immature sperm cells with protoplasmic droplet, head defects, defects of midpieces, tail defects, and defects of acrosome [20].

### Statistical analysis

The software used to perform the statistical analysis was the Statistica 12 software (StatSoft CR s.r.o., Prague, Czech Republic). Data was analysed via analysis of variance procedure, two-way analysis of variance of main effects (post hoc analysis using the Tukey test). The effects of the time of storage (time), a choice of extender (extenders) and interaction between time of storage and extenders were included in the following equation:


$$Y_{ij}=\mu +{time}_{i}+{extenders}_{j}+{interaction}_{ij}+e_{ij}$$

The data in following tables is expressed as the mean±standard error of the mean. The differences were considered statistically significant at p<0.01.

## RESULTS

Quantitative parameters of the native ejaculate of stallions reached high average values, especially the total motility, which was 93.79% (Table 1) and progressive motility which was 89.73%. The average sperm viability of the native ejaculate reached 72.94% and the percentage of spermatozoa of normal morphology was 76.79%.

Main effects (time of storage and extenders) were statistically significant (p<0.01) for all evaluated parameters (motility, progressive motility, viability, and morphology). The interaction of the main effects (time of storage and extenders) was statistically non-significant.

Quantitative parameters of diluted ejaculates decline over time of storage (Table 2). Values of total motility, progressive motility and viability differed statistically highly significant in all time intervals (p<0.01). Values of sperm with normal morphology differ statistically highly significant between 24- and 48-hour time interval (p<0.01) and statistically highly significant between 24- and 72-hour time interval (p<0.01). There was no statistically significant difference in values of sperm with normal morphology between 48- and 72-hour time interval.

The total motility, progressive motility, and values of sperm with normal morphology were significantly higher in semen samples diluted with INRA 96 extender and BotuSemen Gold extender than in samples diluted with SM (p<0.01, Table 3). The sperm viability was significantly highest in semen samples diluted with INRA 96 extender (p<0.01), exceeding BotuSemen Gold and SMs. Sperm viability of samples diluted with BotuSemen Gold extender was significantly higher than viability of samples diluted with SM (p<0.01).

**Figure 1:**
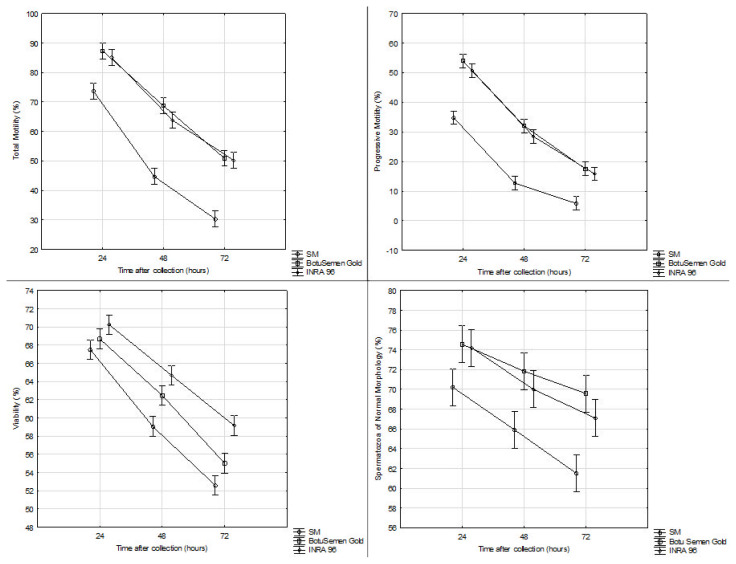
Quality parameters (means±standard error of the mean) of stallion semen samples diluted with selected extenders in different time intervals after collection.

The total motility of semen cooled with the INRA 96 extender was higher than that of the semen cooled with the SM by 11.46% after 24 hours, by 19.07% after 48 hours and by 19.96% after 72 hours (Figure 1). The total motility was also higher with BotuSemen Gold when compared with the SM extender, namely by 13.67% after 24 hours, by 24.02% after 48 hours and by 20.63% after 72 hours. The progressive motility was higher with INRA 96 than with the SM by 15.79% after 24 hours, by 15.75% after 48 hours and by 9.96% after 72 hours. The progressive motility was also higher with the BotuSemen Gold extender than with the SM, namely by 19.20% after 24 hours, by 19.33% after 48 hours and by 11.64% after 72 hours.

On average, BotuSemen Gold maintained slightly higher motility values than INRA 96 at each time interval. Differences within the range of 0.67% to 4.95% in the total motility and 1.68% to 3.58% in the progressive motility were also found between the two extenders.

After 24 hours of storage, the average values of sperm viability between the individual extenders differ only slightly. After 48 hours, the viability was higher by 5.63% with the INRA 96 extender than with the SM extender. The difference between the INRA 96 and BotuSemen Gold extenders was minimal within this period. After 72 hours, the viability was higher by 6.59% with INRA 96 than with the SM and by 4.16% with INRA 96 than with the BotuSemen Gold extender. After 48 and 72 hours, the BotuSemen Gold extender preserved the viability better than SM extender by 3.42% and 2.43%.

Samples with chemically defined extenders INRA 96 and BotuSemen Gold maintained higher average values of sperm with normal morphology than the SM extender. After 24 hours, it was 4.01% to 4.36% higher, after 48 hours, it was 4.12% to 5.94%. After 72 hours, the samples with the INRA 96 extender maintained values by 5.62% higher than the SM extender. After 72 hours, the share of spermatozoa of normal morphology was higher with the BotuSemen Gold extender than with the SM extender by 8.10%. On average, the BotuSemen Gold extender maintained the highest values of sperm with normal morphology in all monitored time intervals; the differences between it and INRA 96, however, were minimal.

## DISCUSSION

The quality of insemination doses is influenced by several factors such as the share of seminal plasma, the ejaculate cooling rate, storage temperature and the extender used [3, 21–23]. The composition of an extender is one of the key factors affecting the ejaculate quality [3,9]. Cooled insemination doses of stallions are kept at the temperature of 4°C to 6°C and used within 24 hours after the ejaculate collection [24]. In our study, we have evaluated the samples of native and diluted ejaculates, using different types of extenders. Samples were kept at a temperature of 4°C and regularly evaluated up to 72 hours after collection.

Results of the study have shown that the average values of the sperm motility and morphology of the native ejaculate had met the requirements. The native ejaculate should contain 60% of motile spermatozoa and 60% of spermatozoa of normal morphology. Věžník et al [20] recorded the average value of stallion sperm viability in native ejaculate being 77.3% ±4.92%. Merkies et al [25] state the viability being within the range from 62% to 78%. Our recorded value was 72.94%± 1.53%. The difference in observed values may be caused by several internal and external factors that influence the quality of an ejaculate [26].

A whole range of extenders allowing short-term preservation of stallions’ ejaculate exists [6]. In our study, we focused on the commonly used SM and some of the best commercially available extenders – INRA 96 and BotuSemen Gold [3,12]. Advantages of SM are the low cost [17], practical usage and effectiveness [10]. The issue with the SM lies in the complex composition of milk as a biological product, the absence of standardization possibilities and possible variation between individual batches. These factors affect the spermatozoa viability and quality of insemination doses [6,9,10,27]. Thus, current efforts focus on the replacement of non-defined components of extenders with components of exact and unchanging composition [5,9]. INRA 96 and BotuSemen Gold belong in this group of extenders [12]. INRA 96 contains modified Hanks’ salts, glucose, and lactose, along with native phosphocaseinate [6], and has become the world’s superior and most widely used extender for equine insemination. However, the results of a new study have shown that BotuSemen Gold, containing sodium caseinate combined with cholesterol-loaded cyclodextrin, can be a suitable alternative to INRA 96 and, in some qualitative parameters, it even achieves better results [12].

The efficiency of extenders was evaluated based on the total and progressive motility, viability, and morphology of sperm. Parameters of sperm motility and morphology are within the basic methods of evaluation of sperm quality [28]. Despite this fact, more detailed tests such as evaluation of the plasma membrane integrity – viability – are necessary [28].

Quality of insemination dose decreases during storage [3,12,29]. In our research, the most significant decline occurred in the motility and the viability parameters.

Results have shown that the ejaculate samples containing commercially available extenders with chemically defined compositions, INRA 96 and BotuSemen Gold, had higher parameters of total and progressive motility (p<0.01) than the SM. LeFrapper et al [3] recorded similar results when comparing the progressive motility between extenders with a specified composition containing caseinates and milk-based extenders [3]. Within our study, the BotuSemen Gold extender maintained, on average, better motility values than INRA 96. The results, however, were not statistically significant. Novello et al [12] recorded statistically higher parameters of motility with BotuSemen Gold than with INRA 96. They were most likely caused by adding cholesterol, present in the BotuSemen Gold extender since the native phosphocaseinate and sodium caseinate are similar molecules, ensuring the protection of spermatozoa.

Sperm viability was significantly highest in the INRA 96 extender (p<0.01), exceeding both, BotuSemen Gold and SM. Batellier et al [6] state that INRA 96 protects spermatozoa more effectively than common milk-based extenders during storage. Novello et al [12] have recorded no differences in the integrity of plasmatic membranes between the INRA 96 extender and the BotuSemen Gold extender after 24 and 48 hours. In our study, the highest differences in viability between these extenders occurred exceeding 48 hours of storage.

Sperm morphology was significantly higher in the INRA 96 extender and BotuSemen Gold extender than in the samples diluted with SM (p<0.01). The BotuSemen Gold reached highest average value of sperm with normal morphology. With the SM, there was a higher share of swollen or missing acrosomes. Hartwig et al [15] state that cholesterol-loaded cyclodextrin causes prolongation of sperm capacitation, which may cause the difference between extenders.

Within our study, all quantitative parameters declined over time, with most significant differences in motility and viability parameters. Extenders with chemically defined compositions had demonstrably better parameters of sperm motility and morphology than the SM. INRA 96 preserved sperm viability more effectively than SM and even better than the BotuSemen Gold extender.

## CONCLUSION

Extenders with chemically defined compositions preserved the ejaculate’s quality better than the milk-based extender. Statistical differences between INRA 96 and BotuSemen Gold extenders occur only in sperm viability, where INRA 96 preserved sperm membrane intactness better than BotuSemen Gold. The highest differences between these extenders occur after 72 hours of storage. Thus, BotuSemen Gold was a suitable alternative to INRA 96 for storage up to the 48 hours. Despite the lowest values of evaluated parameters when using milk-based extender, its advantage remains in affordability and practicality.

## Figures and Tables

**Table 1:** Quality parameters (means±standard error of the mean) of native ejaculate

Items	Native ejaculate	n
Total motility (%)	93.79±0.78	57
Progressive motility (%)	89.73±1.23	57
Viability (%)	72.94±1.53	57
Spermatozoa of normal morphology (%)	76.79±1.52	57

n, number of native ejaculate samples.

**Table 2:** Quality parameters (means±standard error of the mean) of stallion semen samples in time intervals of 24, 48, and 72 hours

Items	n	24 h	48 h	72 h
Total motility (%)	57	82.02±1.23^a^	59.03±1.89^b^	43.80±1.88^c^
Progressive motility (%)	57	46.44±1.66^a^	24.38±1.54^b^	13.08±0.99^c^
Viability (%)	57	68.81±0.58^a^	62.06±0.64^b^	55.58±0.71^c^
Spermatozoa of normal morphology (%)	57	72.99±1.04^a^	69.24±1.13^b^	66.05±1.10^b^

a–c Differences are significant at p<0.01.

**Table 3:** Quality parameters (means±standard error of the mean) of stallion semen samples for each extender

Items	n	Skimmed milk-based extender	Inra 96	BotuSemen Gold
Total motility (%)	57	49.53±2.00^a^	66.36±2.03^b^	68.97±1.88^b^
Progressive motility (%)	57	17.78±1.41^a^	31.62±1.79^b^	34.51±1.82^b^
Viability (%)	57	59.70±0.81^a^	64.69±0.67^c^	62.05±0.76^b^
Spermatozoa of normal morphology (%)	57	65.86±1.16^a^	70.44±1.13^b^	71.99±0.98^b^

a–c Differences are significant at p<0.01.
